# Prevalence of depression among oocyte donor candidates: a systematic review and meta-analysis

**DOI:** 10.1097/MS9.0000000000004523

**Published:** 2025-12-16

**Authors:** Pegah Rashidian, Ehsan Amini-Salehi, Sepide Ahmadi, Seyyed Amirhossein Salehi

**Affiliations:** a Vali-e-Asr Reproductive Health Research Center, Family Health Research Institute, Tehran University of Medical Sciences, Tehran, Iran; b School of Medicine, Guilan university of Medical Science, Rasht, Iran; c Student Research Committee, School of Medicine, Shahid Beheshti University of Medical Sciences, Tehran, Iran

**Keywords:** assisted reproductive technology, depression, donor, oocyte donation, pregnancy

## Abstract

**Background:**

Infertility, affecting approximately 17.5% of individuals worldwide, is increasing globally, making oocyte donation an important reproductive option. However, it is associated with physical and psychological risks, as well as social stigma. The primary aim of this systematic review and meta-analysis is to estimate the prevalence of depression among women undergoing oocyte donation, and the secondary aim is to assess changes in depressive symptoms before and after the donation process.

**Methods:**

A systematic and thorough literature search was performed from 1 January 1990 to 31 December 2024 using PubMed, Web of Science, Scopus, and Embase databases. Reference lists of relevant articles, as well as Google Scholar, were manually reviewed to identify additional eligible studies. The risk of bias of included studies was independently assessed using the Hoy checklist for prevalence studies. Meta-analyses were conducted utilizing STATA version 18.0, applying a random-effects model to account for potential heterogeneity among studies.

**Results:**

The meta-analysis of five studies estimated a pooled depression prevalence of 2% [95% confidence interval (CI): 0–6%; *I*^2^ = 57.7%] among women considered for oocyte donation. No publication bias was detected, and meta-regression showed no significant association between age and depression prevalence (*P* = 0.84). Additionally, no significant change in depression scores was observed before and after donation (Hedge’s *g* = −0.09, 95% CI: −1.92 to 1.74; *I*^2^ = 3.3%).

**Conclusions:**

Depression affects a small but notable proportion of women undergoing oocyte donation, with no significant changes in depressive symptoms before and after the procedure.

## Introduction

Infertility is a significant and growing global health concern with a lifetime prevalence of 17.5%^[1,2]^, and by 2050, over 75% of countries are projected to have fertility rates too low to sustain population growth^[3]^. Among those affected by infertility, approximately 28% are unable to use their own genetic material for reproduction^[4]^. For these individuals, oocyte donation has become a vital reproductive solution. The practice involves using gametes donated by healthy, young women and is indicated in cases of diminished ovarian reserve, premature ovarian insufficiency or menopause, recurrent *in vitro* fertilization (IVF) failure, poor oocyte or embryo quality, heritable genetic diseases, and conditions such as Turner syndrome^[5–7]^. Since the first successful gamete donation in 1984, this approach has become a widely accepted and commonly employed assisted reproductive method, offering one of the few viable paths to biological parenthood for affected individuals^[8]^.

**HIGHLIGHTS**

- Depression affects ~2% of women undergoing oocyte donation.
- No significant change in depression before vs. after oocyte donation.
- No link found between age and depression in oocyte donors.
- Findings support need for psychological support during donation process.

However, oocyte donation carries notable physical and psychological risks for donors. The process requires hormonal stimulation, frequent transvaginal ultrasound monitoring, and surgical retrieval of oocytes, all of which can be physically demanding and emotionally stressful^[9]^. Reported physical complications include ovarian hyperstimulation syndrome (OHSS), infection, and ovarian torsion^[10]^. Additionally, the pharmacologic agents used in ovarian stimulation, including gonadotropins and gonadotropin-releasing hormone agonists, are associated with a range of psychiatric side effects such as mood lability, irritability, anxiety, and depressive symptoms^[11–13]^. Epidemiological and clinical evidence suggests that some women are particularly vulnerable to developing or relapsing into psychiatric symptoms during periods of hormonal fluctuation^[14]^, placing oocyte donors at elevated risk for mental health deterioration during and after the donation process.

Psychological stress may be further intensified by socio-cultural factors. In many traditional societies, oocyte donation remains stigmatized, subjecting donors to ethical concerns, social judgment, and religious tension^[15]^. These pressures can add to the psychosocial burden of the donation process and potentially contribute to adverse mental health outcomes, including anxiety and depression. Beyond the immediate well-being of the donor, important ethical and genetic considerations arise. Depression has a known heritable component^[16,17]^. Studies have also shown that major depressive disorder is linked to mitochondrial dysfunction, which can affect cellular energy production and neural function^[18–21]^. Because both nuclear and mitochondrial deoxyribonucleic acid are passed from the donor to the offspring, assessing the donor’s physical and mental health is essential to ensure both donor and offspring well-being^[22]^.

Despite the increasing use of oocyte donation in assisted reproduction, a lack of systematic evaluation of the psychological well-being of donor candidates persists. To the best of our knowledge, this is the first systematic review and meta-analysis to examine the prevalence and risk of depression among women undergoing oocyte donation, as well as changes in depressive symptoms before and after the procedure. By addressing this gap, the study aims to inform donor screening protocols, enhance psychosocial support, and contribute to ethically responsible practices in reproductive medicine.

## Materials and methods

This systematic review and meta-analysis adhered to the methodological standards outlined in the Preferred Reporting Items for Systematic Reviews and Meta-Analyses (PRISMA) statement^[23]^, ensuring transparency and consistency in the reporting process. Furthermore, this systematic review has been conducted and reported in accordance with the AMSTAR (A Measurement Tool to Assess Systematic Reviews) guidelines^[24]^. This work is also reported in line with the TITAN (Transparency In The reporting of Artificial INtelligence) guidelines^[25]^. No artificial intelligence tools were used in the research or manuscript development.

### Research question

The primary objective of this systematic review was to estimate the prevalence of depression among candidates for oocyte donation. The secondary objective was to assess changes in depression levels before and after the donation process.

### Search strategy

This systematic review employed a comprehensive and structured search strategy to identify relevant literature examining depression among oocyte donor candidates. The search was conducted on 19 January 2025, and covered publications from 1 January 1990 to 31 December 2024. Four major electronic databases – PubMed, Web of Science, Scopus, and Embase – were systematically searched. To enhance the completeness of the search, the reference lists of all included studies were manually screened, and an additional search was performed using Google Scholar to identify any potentially eligible articles not captured in the initial database queries. The search was restricted to articles published in English. Search terms were developed to capture two core concepts: oocyte donation and depression. Keywords, Boolean operators, and database-specific filters were tailored to each platform. The detailed search strategies for all databases are presented in Supplemental Digital Content 1, available at: http://links.lww.com/MS9/B53. Studies eligible for inclusion comprised observational and descriptive research conducted in any geographic setting. Additionally, non-observational studies were considered if they provided data relevant to the primary outcome.

### Inclusion and exclusion criteria

Studies were considered eligible for inclusion if they investigated the prevalence of depression among oocyte donor candidates, reported sufficient data to allow for the calculation of depression prevalence, or assessed changes in depression levels before and after oocyte donation. Eligible studies could use standardized depression assessment tools that provided depression scores or report the number of participants exhibiting depressive symptoms.

Studies were excluded if they did not address the primary outcome of interest, failed to provide adequate data for analysis, or involved non-human subjects. In addition, the following publication types were excluded: review articles, case reports, case series, brief communications, meeting abstracts, book chapters, letters, editorials, commentaries, correspondence, and study protocols.

### Study selection

The screening process was carried out independently by two reviewers, who first evaluated the titles and abstracts of all retrieved records to determine their potential relevance. Full-text articles were subsequently reviewed in detail to assess eligibility according to the predefined inclusion and exclusion criteria. Disagreements between the two reviewers were resolved through discussion. If a consensus could not be achieved, a third reviewer was consulted to facilitate resolution.

### Data collection

Data were independently extracted by two reviewers using a standardized approach. Any discrepancies in the extracted information were resolved through discussion, and a third reviewer was consulted when necessary to ensure consistency and accuracy. The extracted data were systematically categorized into three domains: (1) general study characteristics, including first author, year of publication, country, and study design; (2) participant-related data, such as the total number of oocyte donor candidates, number of individuals with and without depression before and after donation, and participant age; and (3) depression-specific information, including the assessment instruments employed and the mean depression scores reported pre- and post-donation.

### Quality assessment

The methodological quality of each included study was evaluated by two independent reviewers using a 10-item checklist developed by Hoy *et al*^[26]^, specifically designed to assess the risk of bias in prevalence studies. Any disagreements between reviewers regarding the risk of bias were resolved through discussion, with a third reviewer consulted when necessary to reach a final consensus. The checklist covers four items related to external validity (e.g., sample representativeness and random selection) and six items related to internal validity (e.g., clarity of case definitions and consistency of data collection). Each item on the checklist was scored as “Yes” (1 point, indicating low risk of bias) or “No/Unclear” (0 points, indicating high risk of bias or insufficient information). The total score for each study was then calculated and used to categorize studies as follows: (1) low risk of bias (7–10 points), (2) moderate risk of bias (4–6 points), and (3) high risk of bias (1–3 points). For example, a study scoring 8 “Yes” responses and 2 “No” or “Unclear” responses would be classified as low risk of bias, reflecting adequate methodological quality.

### Meta-analysis

The data extracted from eligible studies were initially organized using Microsoft Excel and subsequently imported into STATA MP 18 statistical software (Stata Corp LLC, College Station, TX, USA) for statistical analysis. The primary objective of the meta-analysis was to estimate the prevalence of depression among oocyte donor candidates. The prevalence was expressed as a proportion (percentage) with corresponding 95% confidence intervals (CIs). To assess the presence of heterogeneity across the studies, both the Cochrane *Q* test and the *I*^2^ statistic were employed. A value of *I*^2^ greater than 50% was considered indicative of substantial heterogeneity. The Cochrane *Q* test evaluates whether observed differences in results across studies are compatible with chance alone, while the *I*^2^ statistic quantifies the proportion of total variation in effect estimates that is due to heterogeneity rather than sampling error, with higher values indicating greater heterogeneity. A Galbraith plot was created to assess the variation in individual study estimates within the 95% CI range, helping to identify outliers or significant deviations from the overall trend. A random-effects model using the restricted maximum likelihood (REML) method was applied to pool the depression prevalence across studies. The REML method estimates the between-study variance and provides more accurate pooled estimates, particularly when heterogeneity is present, making it suitable for meta-analyses with varying study populations. This model was selected due to the anticipated variability in study populations and exposure factors. Sensitivity analysis was conducted using a leave-one-out approach to examine the robustness of the results by excluding each study in turn. Two subgroup analyses were performed to explore potential sources of heterogeneity. The first subgroup analysis was based on the risk of bias classification of the studies, and the second analyzed the studies by their country of origin. Publication bias was evaluated through both qualitative and quantitative methods. A contour-enhanced funnel plot was used for visual inspection of asymmetry, while Begg’s^[27]^ nonparametric rank correlation test and Egger’s^[28]^ regression test were employed to statistically detect small-study effects. A *P*-value below 0.05 in these tests indicated a potential for publication bias. To address any such bias, Duval and Tweedie’s trim-and-fill method^[29]^ was applied to adjust the pooled estimates accordingly.

In addition to the primary analysis, a random-effects model was also used to assess the pooled changes in depression scores before and after oocyte donation. Hedge’s *g* was calculated to estimate the effect size for comparing the mean differences between the two groups, with 95% CIs provided for the results.

Finally, a meta-regression analysis was conducted to investigate potential sources of heterogeneity, with age as the primary covariate. This analysis sought to determine whether age was a significant factor influencing variations in depression prevalence among oocyte donors.

## Results

### Search results

**Figure 1.**
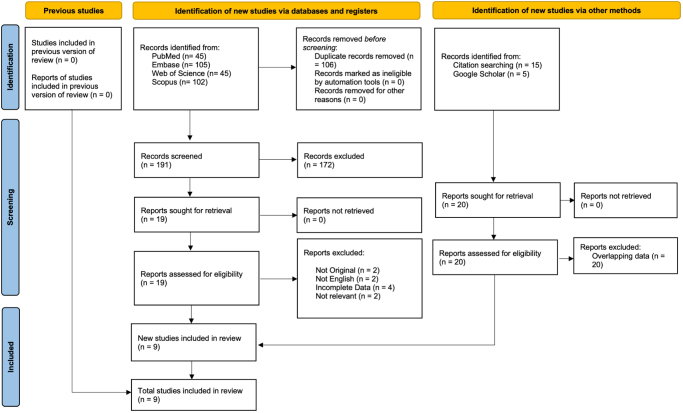
PRISMA 2020 flow chart.

A total of 297 records were identified through the systematic search. After the removal of duplicates, 191 records remained for screening of titles and abstracts. Following this initial screening, 172 records were excluded based on the predefined inclusion and exclusion criteria. The full texts of the remaining 19 records were then assessed for eligibility, leading to the inclusion of 9 articles that met the inclusion criteria. Overall, the included studies were generally of low to moderate risk of bias, with most studies assessed as low risk (see the “Quality assessment” section in the Results section for details). Manual screening of the reference lists from the included studies and a search of the Google Scholar database did not yield any additional eligible articles. A flowchart of the study selection process, following the PRISMA 2020 guidelines, is presented in Figure 1, and a table detailing the excluded studies is provided in Supplemental Digital Content 1, Table S1, available at: http://links.lww.com/MS9/B53.

### Study characteristics

Nine studies^[13,30–37]^ met the inclusion criteria and were incorporated into the systematic review, with sample sizes ranging from 13 to 170 participants. The included studies were published between 1995 and 2023 and were conducted across diverse geographic regions, including Asia, Europe, and North America. The most commonly employed study design was cross-sectional, followed by prospective cohort designs. One randomized controlled trial^[35]^ was also included, as it provided relevant data for the analysis.

Of the nine studies, seven^[30–34,36,37]^ reported data suitable for estimating the prevalence of depression among oocyte donor candidates. However, two of these^[33,37]^ were follow-up reports from the same cohort^[32]^ and were therefore excluded from the meta-analysis to avoid duplication of data. Two studies^[13,35]^ provided quantitative data on depression scores both before and after oocyte donation and were included in a separate meta-analysis to assess changes in depression levels following oocyte retrieval.

Additionally, one study^[33]^ reported the change in the number of participants classified as depressed before and after donation. Due to its unique format, this study was reviewed but excluded from the meta-analytical pooling. A comprehensive summary of the characteristics of the included studies is presented in Table 1. Additionally, the funding sources of all included studies are presented in Supplemental Digital Content 2, Table S2 available at: http://links.lww.com/MS9/B54.

**Table 1:** Characteristics of the included studies

**Author (year)**	**Country**	**Study design**	**Mean age ± SD or median (range), y**	**Total OD candidates**	**Questionary**	**Number of women who were depressed based on the questionary**	**Number of women who were not depressed based on the questionary**	**Study quality (HOY)**
Lindheim (1995)^[28]^	USA	CS	29.7 ± 1.4	13	N/A	0	13	Moderate ROB
Williams (2011)^[29]^	USA	CS	28.0 ± 4.9	28	POMS/BDI-ii	0	28	Low ROB
Svanberg (2012)^[30]^	Sweden	CS	30.4 ± 4.43	170	HADS	3	167	Low ROB
Svanberg (2013)^[31]^	Sweden	PC	30.39 ± 4.53	161	HADS	3	158	Low ROB
Makvandi (2018)^[33]^	Iran	CS	N/A	75	SCID	1	74	Low ROB
Sharafi (2023)^[34]^	Iran	CS	28.79 ± 4.1	39	N/A	5	34	Moderate ROB
Sydsjö (2023)^[35]^	Sweden	PC	46 (35−56)	121	HADS	2	119	Low ROB
Kazemi (2016)^[32]^	Iran	PC	28.36 ± 3.28	63	GHQ-28	5.05 ± 5.1	4.03 ± 5.1	Low ROB
Adib Moghaddam (2023)^[13]^	Iran	RCT	28.94 ± 3.30	36	DASS- 21	3.77 ± 3.57	4.16 ± 4.17	Low ROB

BDI-ii: Beck Depression Inventory-II; CS: Cross-Section; DASS: Depression Anxiety Stress Scales; GHQ: General Health Questionnaire; HADS: Hospital Anxiety and Depressions Scale; N/A: not available; OD: oocyte donation; PC: prospective cohort; POMS: profile of mood states; RCT: randomized controlled trials; ROB: risk of bias; SCID: structured clinical interview for DSM disorders; SD: standard deviation; y: years.

### Quality assessment

The methodological appraisal of the included studies indicated that seven^[13,31–35,37]^ were assessed as having a low risk of bias, while the remaining two^[30,36]^ were deemed to have a moderate risk of bias. A comprehensive summary of the quality assessment outcomes is available in Supplemental Digital Content 2, Table S3, available at: http://links.lww.com/MS9/B54.

The primary sources of potential bias were related to aspects of external validity, including limitations in the representativeness of the study populations, insufficiently defined sampling frames, and non-randomized or unclear case selection procedures. In contrast, internal validity was generally well maintained across the included studies, with consistent methodologies and clearly defined case criteria.

### Meta-analysis

#### Prevalence of depression among oocyte donor candidates

A meta-analysis was performed using data from five studies^[30–32,34,36]^ that reported the prevalence of depression among individuals being considered as oocyte donors. The pooled prevalence was estimated at 2% (95% CI: 0–6%; *P* = 0.07), with moderate heterogeneity detected among studies (*I*^2^ = 57.68%, indicating that approximately 58% of the variability in prevalence estimates was due to differences between studies rather than chance). Analysis using a Galbraith plot identified one study as an outlier, reporting a higher prevalence than the others, which may reflect differences in study population characteristics, assessment methods, or cultural context. Despite this, leave-one-out sensitivity analysis revealed that the overall prevalence estimate remained stable, varying between 1% and 3%, indicating that no single study had an undue influence on the overall results.

To investigate potential sources of heterogeneity, subgroup analyses were conducted. When stratified by risk of bias, studies rated as having low risk of bias yielded a pooled prevalence of 1% (95% CI: 0–3%; *I*^2^ = 0.00%; *P* = 0.02), whereas those with moderate risk of bias showed a higher prevalence of 6% (95% CI: 0–23%; *I*^2^ = 58.35%; *P* = 0.15). Country-based subgroup analysis revealed variability in depression prevalence: studies conducted in Iran reported a pooled rate of 5%, those from Sweden 2%, and the study from the USA reported 0%.

**Figure 2.**
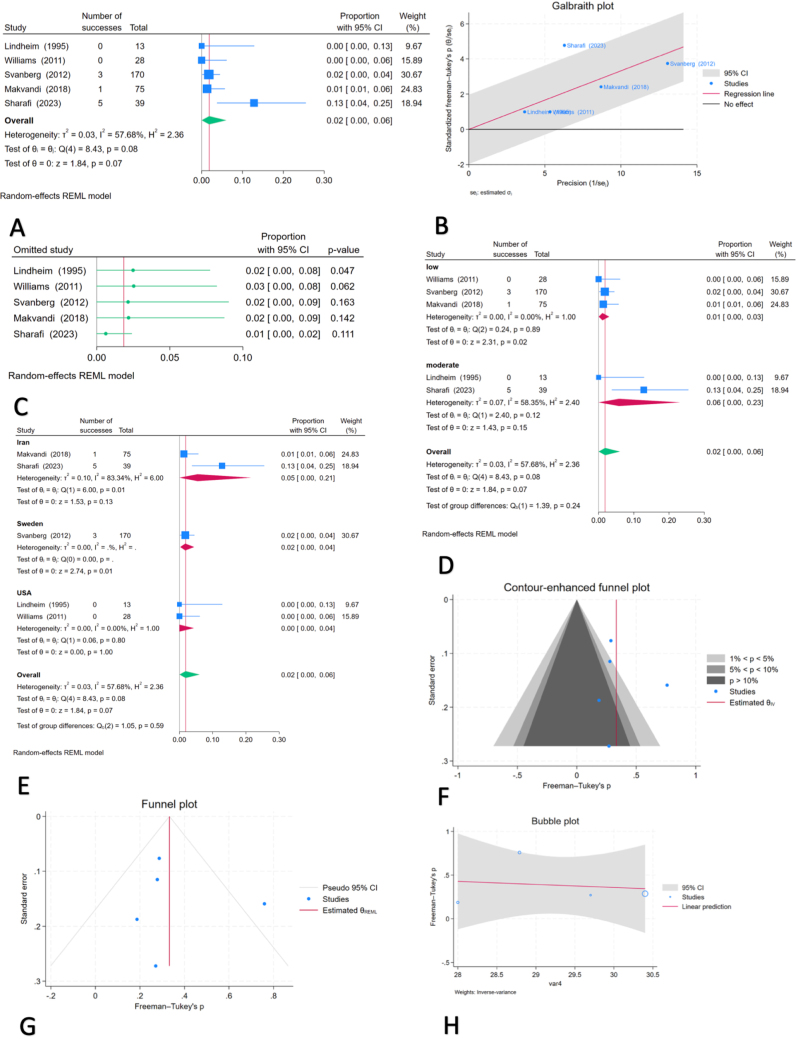
Findings of the meta-analysis of five studies estimating the prevalence of depression among oocyte donor candidates: (A) forest plot, (B) Galbraith plot, (C) leave-one-out sensitivity analysis, (D) subgroup analysis of pooled prevalence rates by risk of bias, (E) Subgroup analysis of pooled prevalence rates by country of origin, (F) contour-enhanced funnel plot, (G) funnel plot with the trim-and-fill method, and (H) bubble plot from meta-regression analysis.

Assessment of publication bias using Egger’s regression test (*P* = 0.93) and Begg’s rank correlation test (*P* = 0.46) did not indicate the presence of small-study effects. These findings were further supported by visual inspection of the contour-enhanced funnel plot. No additional studies were imputed using Duval and Tweedie’s trim-and-fill method (Fig. 2).

#### Meta-regression

To explore whether age influenced the prevalence of depression among oocyte donor candidates, a meta-regression was conducted using age as a covariate. Although the analysis revealed a non-significant trend toward decreasing depression prevalence with increasing age, the association did not reach statistical significance (*P* = 0.84). The analysis also indicated considerable residual heterogeneity across studies (*I*^2^ = 69.19%, suggesting that nearly 70% of the variation in study results was due to real differences rather than sampling error), suggesting that other unmeasured factors may be contributing to the variability in prevalence estimates. The results of this analysis are illustrated in Figure 2.

#### Change in depression scores following oocyte donation

**Figure 3.**
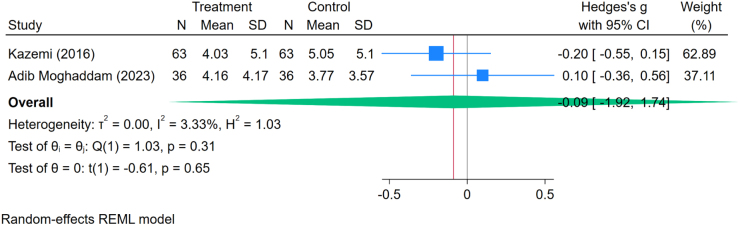
Forest plot of the standardized mean difference in depression scores before and after oocyte donation.

Two studies^[13,35]^ provided sufficient data to evaluate changes in depression levels before and after oocyte retrieval. A random-effects meta-analysis estimated the standardized mean difference using Hedge’s *g* at −0.09 (95% CI: −1.92 to 1.74; *P* = 0.65), suggesting no statistically significant change in depression scores pre- and post-donation. Heterogeneity was minimal (*I*^2^ = 3.33%), indicating that nearly all of the observed variation was due to chance, reflecting high consistency across studies (Fig. 3).

## Discussion

In this systematic review and meta-analysis, we aimed to determine the prevalence of depression among oocyte donor candidates and examine whether depressive symptoms changed throughout the donation process. Our meta-analysis revealed an overall depression prevalence of 2%, with moderate heterogeneity across studies. Subgroup analysis by country and risk of bias indicated variability in prevalence rates, highlighting potential influences of geographic and methodological differences. Importantly, meta-regression did not find a significant association between donor age and depression prevalence.

When evaluating changes in depression over time, the two studies included in our meta-analysis showed minimal to no change in depressive symptoms before and after the donation process. These results suggest that, on average, oocyte donation does not significantly impact donor emotional well-being in terms of depression. However, limited longitudinal data warrant cautious interpretation. Although overall prevalence is low, depression remains a clinically relevant concern due to the emotional and physical stressors associated with the donation process.

Our study has several strengths. It is the first systematic review and meta-analysis, to our knowledge, that quantifies both the prevalence and trajectory of depression among oocyte donors. We conducted a comprehensive literature search across major databases and adhered strictly to PRISMA guidelines, ensuring methodological rigor and transparency. Quality assessment using a validated checklist strengthened the reliability of the included data. Our statistical approach, including random-effects models and sensitivity analyses, supports the robustness of our findings. Nonetheless, limitations must be acknowledged. At the study level, the included studies exhibited moderate heterogeneity in pooled prevalence estimates, reflecting variations in study populations, methodologies, and cultural contexts, which may influence depression prevalence but could not be fully addressed in meta-regression. Only two studies reported longitudinal changes in depressive symptoms before and after oocyte donation, limiting the ability to draw robust conclusions about temporal effects. Furthermore, most studies were conducted in a limited number of countries (e.g., Iran, Sweden, and the USA), restricting the generalizability of the findings. At the review level, the restriction to English-language publications may have introduced selection bias. The relatively small number of included studies could have reduced the statistical power of the meta-analysis and increased susceptibility to publication bias, although formal tests did not indicate significant bias. Additionally, inherent limitations in the available literature, such as heterogeneity in study designs and outcome assessments, constrained the depth of our analyses.

Our findings align with previous research suggesting that oocyte donors generally maintain stable psychological health throughout the donation process. For example, the meta-analysis reported a standardized mean difference in depression scores using Hedge’s *g* at −0.09 (95% CI: −1.92 to 1.74), indicating no substantial change in depressive symptoms. The low heterogeneity (*I*^2^ = 3.33%) across these studies supports the consistency of these findings.

Svanberg *et al*^[33]^, in a study that was also included in our meta-analysis, found a slight increase in depression prevalence post-donation – from 1.9% (3 out of 161 donors) to 4.3% (7 out of 163 donors) – although the majority of donors did not report significant depressive symptoms.

Similarly, a long-term follow-up study by Gunilla Sydsjö *et al*^[37]^ found an increase in depression prevalence from 2% at time of donor acceptance (2 out of 119) to 7% during a 14–17-year follow-up (9 out of 123). However, most donors remained psychologically well, and overall satisfaction with the donation experience was high: 96% felt they had contributed to fellow human beings, 98% were happy to help couples unable to conceive, and 93% felt more content after donation.

Compared to oocyte donors, women undergoing IVF for their own fertility report markedly higher depression rates – 20–50% for mild to moderate depression and around 2% for severe depression – especially after failed cycles^[38,39]^. In contrast, oocyte donors seem to experience lower depression rates, likely due to differing stressors. The sense of altruism reported by many donors^[37,40]^ may serve as a protective factor.

Differences in depression prevalence by country are also important. Studies from Iran reported a pooled prevalence of 5%, compared to lower rates in Sweden and the USA. Religious and social stigma may explain this discrepancy. According to Ghelich-Khani *et al*^[41]^, Islamic teachings, particularly around the concept of *mahram* (genetic lineage and family relationships), may contribute to psychological distress in Iranian donors, who fear disclosure and judgment.

The low overall prevalence of depression among oocyte donors is encouraging, but it should not lead to complacency in clinical settings. Oocyte donation involves invasive procedures (e.g., ovulation induction, repeated ultrasounds, and anesthesia)^[9,10,42]^, and donors may experience physical side effects such as OHSS, infection, or ovarian torsion^[43]^. These physical stressors, coupled with potential psychological symptoms (e.g., irritability, mood swings, and depressed mood)^[11]^, create a vulnerable period.

Donors also face complex emotional decisions regarding anonymity and future relationships with donor-conceived children^[44]^, particularly when donating to known recipients such as friends or relatives^[15]^. While most donors describe their experience as positive^[40]^, some report post-donation sadness or regret^[45]^. These findings underscore the importance of comprehensive psychological support throughout the donation process.

Clinicians should incorporate routine mental health screening, ensure pre- and post-donation counseling, and tailor support to cultural context – particularly in settings with higher stigma. Additional research is required before generalizing these findings to all clinical settings.

This study highlights several areas for future research. Longitudinal studies are needed to explore long-term mental health outcomes in oocyte donors, as only limited follow-up data currently exist^[33,37]^. Additionally, identifying factors that confer psychological resilience (e.g., altruistic motives) versus risk (e.g., social stigma and inadequate support) could improve donor care. Further research should examine the effectiveness of mental health interventions or psychosocial support services within oocyte donation programs. Studies from underrepresented regions and non-English publications should also be included in future reviews to broaden generalizability and cultural understanding. Meta-regressions incorporating a wider range of potential confounders – such as prior psychiatric history, cultural factors, and donation protocols – would refine our understanding of depression risk in this population.

In conclusion, our systematic review and meta-analysis provide insights into the prevalence of depression among oocyte donor candidates and its changes throughout the donation process. The overall prevalence of depression was found to be relatively low at 2%, although there was some variability across studies. While our study indicates that the donation process generally does not significantly affect donors’ emotional well-being, reports of increased depressive symptoms in some follow-up studies underscore the need for individualized psychosocial support. Certain geographical regions, such as Iran, showed higher depression rates, highlighting the potential influence of cultural and social factors. Additionally, while most donors reported satisfaction with their experience, a slight increase in depression prevalence was observed in some follow-up studies. These results underscore the importance of providing comprehensive support to oocyte donors, addressing potential emotional challenges and cultural concerns. Future research should focus on exploring the underlying causes contributing to depression in this population and developing interventions to enhance their mental health and well-being.

## Data Availability

Not applicable.

## Research registration unique identifying number (UIN)

The review protocol was prospectively registered in PROSPERO under registration number CRD420251054913. The full registration is publicly accessible at: https://www.crd.york.ac.uk/prospero/display_record.php?ID=CRD420251054913.
